# Influence of Cholera upon Pregnancy and the Fœtus

**Published:** 1850-04

**Authors:** 


*Influence of holera Cupon Pregancy and the Fœtus.*—In the Gazette Médicale of October 13th, this question is investigated by M. Bouchut. The result of inquiries seems to be that the pregnant state is no safeguard against cholera, and that after the third month abortion generally occurs, if the attack of cholera lasts beyond twenty-four hours. In those more rapid cases, the woman often dies without expulsion of the uterine contents. The effects on the fœtus are generally fatal, even where premature labour occurs, as at the seventh or eighth month; the infant is born asphyxiated. [This we have ourselves witnessed.—*Prov. Med. and Surg. Journal.*]

